# Novel ultrashort-acting benzodiazepine remimazolam lowers shivering threshold in rabbits

**DOI:** 10.3389/fphar.2022.1019114

**Published:** 2022-10-14

**Authors:** Kenji Muroya, Kenta Ueda, Keiichi Wada, Masakazu Kotoda, Takashi Matsukawa

**Affiliations:** ^1^ Department of Anesthesiology, Faculty of Medicine, University of Yamanashi, Yamanashi, Japan; ^2^ Surgical Center, University of Yamanashi Hospital, University of Yamanashi, Yamanashi, Japan

**Keywords:** benzodiazepine, remimazolam, shivering, γ-aminobutyric acid, hypothermia

## Abstract

Shivering after surgery or during therapeutic hypothermia can lead to serious complications, such as myocardial infarction and respiratory failure. Although several anesthetics and opioids are shown to have anti-shivering effects, their sedative and respiratory side effects dampen the usefulness of these drugs for the prevention of shivering. In the present study, we explored the potential of a novel ultrashort-acting benzodiazepine, remimazolam, in the prevention of shivering using a rabbit model of hypothermia. Adult male Japanese white rabbits were anesthetized with isoflurane. The rabbits received saline (control), remimazolam (either 0.1 or 1 mg/kg/h), or remimazolam + flumazenil, a selective γ-aminobutyric acid (GABA) type A receptor antagonist (*n* = 6 each). Thirty minutes after discontinuation of the drugs, cooling was initiated by perfusing 10°C water *via* a plastic tube positioned in the colon until the animal shivered. Core body temperature and hemodynamic and physiological parameters were recorded. Remimazolam at 1 mg/kg/h significantly lowered the core temperature change during shivering (−2.50 ± 0.20°C vs. control: −1.00 ± 0.12°C, *p* = 0.0009). The effect of 1 mg/kg/h remimazolam on the core temperature change was abolished by flumazenil administration (−0.94 ± 0.16°C vs. control: −1.00 ± 0.12°C, *p* = 0.996). Most of the hemodynamic and physiological parameters did not differ significantly among groups during cooling. Remimazolam at a clinically relevant dose successfully suppressed shivering in rabbits *via* the GABA pathway even after its anesthetic effects likely disappeared. Remimazolam may have the potential to prevent shivering in patients undergoing surgery or therapeutic hypothermia.

## Introduction

Humans are homeotherms, and their autonomous thermoregulatory system coordinates to maintain internal body temperature within a very narrow range (Morrison and Nakamura, 2019; Simegn et al., 2021). The thermal interthreshold range—the core temperature range that does not trigger autonomic thermoregulatory responses—in humans is usually as narrow as 0.2–0.4°C (Lopez et al., 1994; De Witte and Sessler, 2002; Sessler, 2008). Once the core temperature falls below the lower limit of the interthreshold range, arteriovenous shunt vasoconstriction occurs as the first-line defense against cold to deliver metabolic heat to the thermal core. The second-line defense is so-called nonshivering thermogenesis, where brown fat metabolism is enhanced to facilitate metabolic heat production. If the core temperature falls farther below the interthreshold range, shivering occurs as the final thermoregulatory defense (De Witte and Sessler, 2002). Shivering is an involuntary and vibratory muscular movement that enhances metabolic heat production. A previous study demonstrated that vigorous shivering could induce up to a 500% increase in metabolic heat production compared with basal levels (Giesbrecht et al., 1994).

As described above, shivering is essentially a protective thermoregulatory system that defends against hypothermia. However, shivering after surgery is often problematic and can lead to other serious complications (De Witte and Sessler, 2002; Kurz, 2008). Postoperative shivering and the accompanying cold sensation are remarkably uncomfortable for surgical patients, often worse than surgical pain (De Witte and Sessler, 2002). Moreover, the oscillatory muscle movement aggravates surgical pain by stretching surgical wounds. Shivering stimulates the sympathetic nervous system and can induce tachycardia, arrhythmia, and hypertension. Shivering increases intraocular (Mahajan et al., 1987) and intracranial (Rosa et al., 1995) pressures, oxygen consumption, and carbon dioxide production (Ciofolo et al., 1989; Just et al., 1992; Lienhart et al., 1992). The increased metabolic, physiological, and hemodynamic burden can be critically harmful in patients with reduced cardiac output or limited respiratory reserve (De Witte and Sessler, 2002). Therefore, preventative strategies for postoperative shivering are vital.

Warming devices are useful to maintain the patient’s body temperature. However, the use of these devices might be limited depending on the type or site of surgery, and they cannot always prevent hypothermia. In addition, postoperative shivering can occur even if the patient’s core temperature is maintained within a normal range as a result of elevated interthreshold range due to surgery-induced inflammatory responses (Frank et al., 2000).

Although several drugs are shown to have inhibitory effects on shivering, most of these drugs are long-acting anesthetics or opioids, thus raising concerns about sedative and respiratory side effects (Guffin et al., 1987; Kurz et al., 1997; Wheelahan et al., 1998; De Witte and Sessler, 2002).

Remimazolam is a novel benzodiazepine and has recently been approved for anesthesia and procedural sedation in many countries (Kilpatrick, 2021). Unlike other benzodiazepines, remimazolam contains a metabolically labile ester moiety that produces its ultrashort–acting profile (Chitilian et al., 2013). The combination of this remimazolam and its reversal agent, flumazenil, renders excellent anesthetic controllability. In addition, remimazolam has minimum inhibitory effects on cardiovascular function. With these clinical advantages, remimazolam has become increasingly popular in everyday practice.

The effects of remimazolam on various physiological functions such as neurocognitive, cardiovascular, and respiratory functions have already been well-studied. However, the effect of remimazolam on thermoregulatory function and its possible anti-shivering effect remain unknown. We previously reported that benzodiazepines lower the shivering threshold (Masamune et al., 2009). Therefore, we hypothesized that remimazolam also has inhibitory effects on shivering and that its ultrashort-acting profile has clinical advantages over conventional benzodiazepines. In the present study, we investigated the possible inhibitory effects of remimazolam on shivering and the underlying mechanism using a rabbit model of shivering.

## Materials and methods

The experiments were conducted in accordance with the National Institutes of Health guidelines for the care and use of laboratory animals. The experimental protocol was reviewed and approved by the University of Yamanashi Animal Care Committee (approved protocol number: A3-3). Animals were euthanized by pentobarbital sodium overdose at the end of the experiment.

### Animals

Male Japanese white rabbits (Kbs:JW, age: 10–16 weeks; weight: approximately 3.0 kg) were purchased from Kitayama Labes (Nagano, Japan). The rabbits were housed at 23 ± 2°C under a 12-h light–dark cycle with free access to standard food and water. The normal daytime core temperature of these rabbits is approximately 39°C. The body mass index, defined as the weight divided by the square of the length from the nose to the root of the tail, of these rabbits is approximately 15 kg/m^2^. All experiments were performed between 10 a.m. and 3 p.m. under normal room light and temperature (23 ± 2°C) conditions. All intravenously-administered solutions were.

### Anesthesia and experimental preparation

Animals were prepared for the experiment as previously described (Okuyama et al., 2003; Masamune et al., 2009). Briefly, the animals were anesthetized by inhalation of a mixture of 3% isoflurane (Viatris Pharmaceuticals Japan, Tokyo, Japan) and 67% nitrous oxide (Chiyoda, Tokyo, Japan) in oxygen (Chiyoda). Each animal’s trachea was then intubated with a 3-mm endotracheal tube, and the animals were subsequently allowed to breathe spontaneously. End-tidal carbon dioxide was continuously monitored using a capnogram (Vamos; Drager Medical, Tokyo, Japan). A 24G catheter was inserted into the marginal ear vein for intravenous administration. Lactated Ringer’s solution (Lactec; Otsuka Pharmaceutical, Tokyo, Japan, maintained at 23 ± 2°C) was intravenously administered at a rate of 3 ml/kg/h throughout the experiment. Another 24G catheter was inserted into a femoral artery for blood gas sampling and hemodynamic monitoring. A thermometer probe was inserted into the esophagus for core temperature monitoring (MGA 3-219; Nihon Kohden, Tokyo, Japan). A plastic tube (Salem Sump; Cardinal Health K.K. Tokyo, Japan) was inserted into the colon for cooling and inducting shivering. The esophageal temperature was maintained at 39.0 ± 1.0°C using a heating blanket until cooling was initiated.

### Drug administration and induction of shivering

**FIGURE 1:**
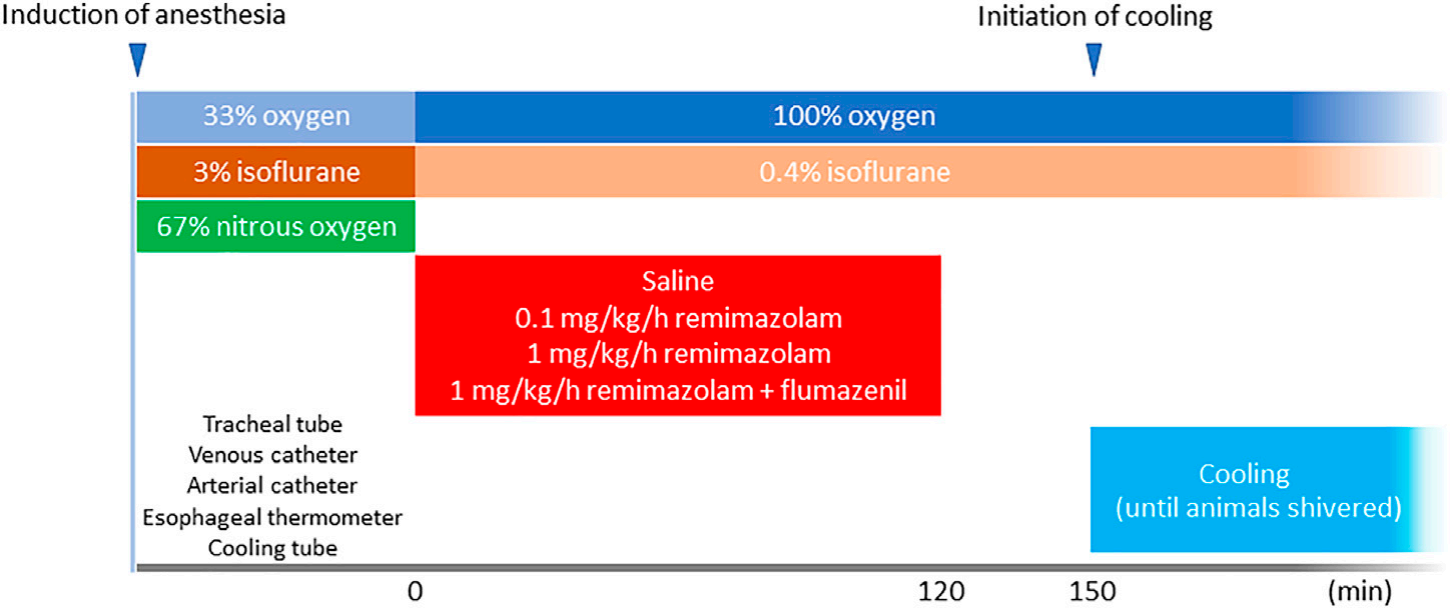
Time flow of the study. A total of 12 animals received either 120-min continuous intravenous administration of saline or remimazolam (either 0.1 or 1 mg/kg/h) (*n* = 6 each). The animals in the remimazolam + flumazenil group animals received 0.01 mg/kg flumazenil at the end of the continuous administration of remimazolam. Cooling was initiated 30 min after the discontinuation of saline/remimazolam administration and continued until the animals shivered.

The animals were randomly allocated into the following groups: saline (control), remimazolam (either 0.1 or 1 mg/kg/h, Mundipharma K.K. Tokyo, Japan), and remimazolam + flumazenil (a selective γ-aminobutyric acid (GABA) type A receptor antagonist; Sawai Pharmaceutical, Osaka, Japan) (*n* = 6 each). Nitrous oxide administration was discontinued and the end-tidal concentration of isoflurane was adjusted to 0.4% in 100% oxygen (NORMAC AA-102; GE Health care, Andover, MA, United States) to minimize the possible influence of volatile anesthetics on the shivering threshold. Rabbits with tracheal intubation are generally well-sedated under 0.4% isoflurane anesthesia and do not present with any signs of noxious tracheal stimulus, such as bucking, moving, and hemodynamic instability. Animals in the remimazolam groups received either 0.1 or 1 mg/kg/h of remimazolam for 120 min; those in the remimazolam + flumazenil group received 1 mg/kg/h of remimazolam for 120 min, followed by a bolus administration of 0.01 mg/kg flumazenil. The control group received the same volume (1 ml/kg/h) of saline for 120 min. The temperature of the solutions was 23 ± 2°C (room temperature). Thirty minutes after discontinuation of the remimazolam/saline administration, cooling was initiated at a rate of 2–3°C/h by perfusing 10°C water *via* the plastic tube positioned in the colon until the animal shivered. Shivering (characteristic tremoring) was identified *via* inspection of the dorsum of the animal by a single trained observer blinded to the grouping. Blood gas analysis was performed before cooling and when the animal shivered. The time flow of this study is shown in Figure 1.

### Statistical analysis

Statistical analysis was conducted using GraphPad Prism 9 (GraphPad Software, San Diego, CA, United States). Ordinary one-way analysis of variance (ANOVA) followed by the Dunnett test was used to analyze the core temperature change and body weight; two-way ANOVA for repeated measures followed by the Dunnett test was used to analyze the hemodynamic and blood gas values. Power analysis indicated that the sample size of 5 animals per group was sufficient to achieve a power of 0.8 with an α level of 0.05 to detect a mean difference of 1°C in core temperature change based on a SD of 0.5°C (G*Power 3.1.9.7). All values are presented as the mean ± SEM. *p* < 0.05 was considered statistically significant.

## Results

**FIGURE 2:**
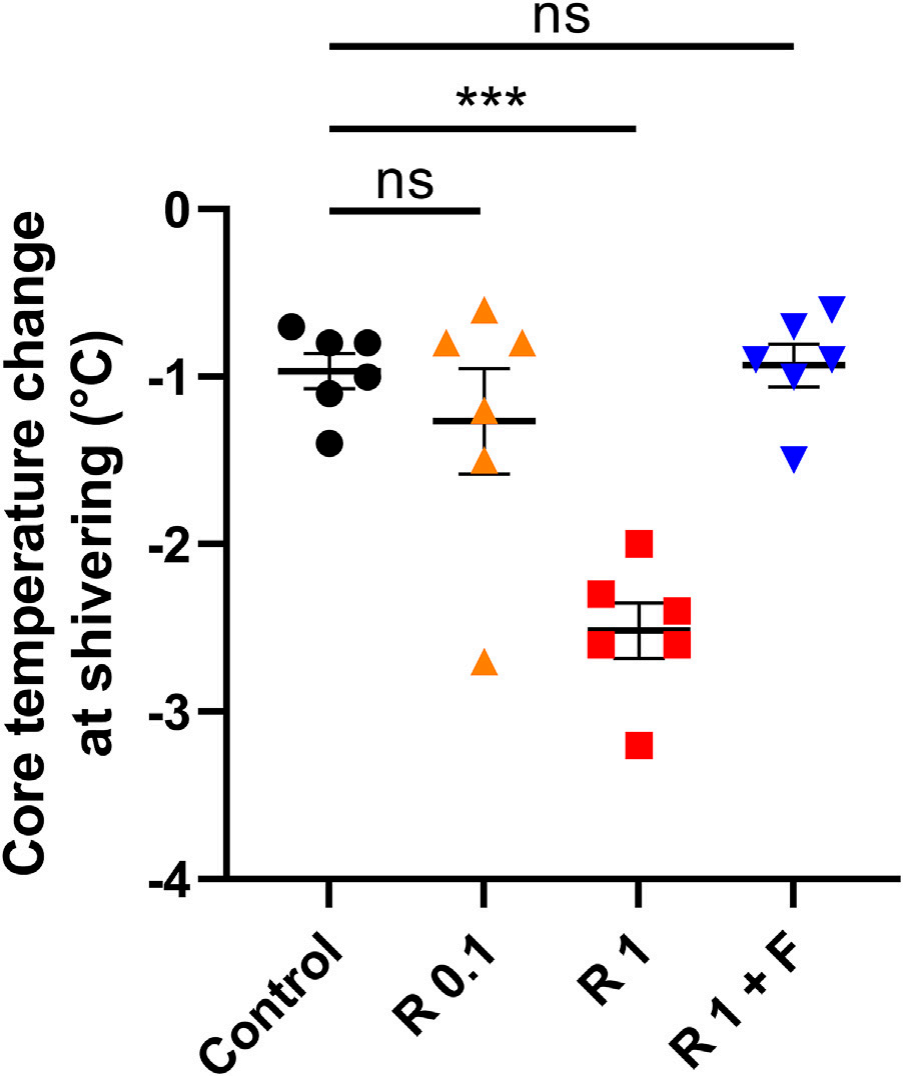
Core temperature change from baseline during shivering. Remimazolam at 1 mg/kg/h significantly lowered the core temperature change during shivering, which was abolished by a bolus flumazenil administration at the end of remimazolam administration (*n* = 6 each). Data are shown as mean ± SEM. ***: *p* < 0.001 R, remimazolam; F, flumazenil.

Remimazolam at 1 mg/kg/h significantly lowered the core temperature change during shivering (−2.52 ± 0.18°C vs. control: −0.97 ± 0.12°C, *p* < 0.001) (Figure 2). Although there was a trend toward lower core temperature changes in rabbits that received remimazolam at 0.1 mg/kg/h compared with that of the control group rabbits, the difference was small and not statistically significant, and the individual difference was large (−1.27 ± 0.35°C vs. control: −0.97 ± 0.12°C, *p* = 0.579). The effect of 1 mg/kg/h remimazolam on the core temperature change was abolished by flumazenil administration (−0.93 ± 0.14°C vs. control: −0.97 ± 0.12°C, *p* = 0.999).

**FIGURE 3:**
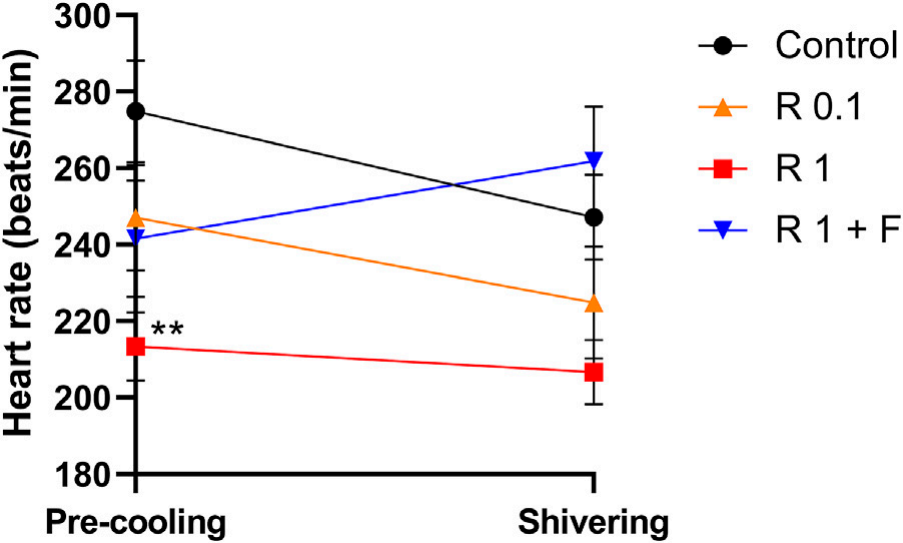
Heart rate before cooling and during shivering. The heart rate before cooling was significantly lower in rabbits that received remimazolam at 1 mg/kg/h compared with the control rabbits (*n* = 6 each). Data are shown as mean ± SEM. **: *p* < 0.01 (vs. control) R, remimazolam; F, flumazenil.

The heart rate of rabbits that received remimazolam at 1 mg/kg/h was significantly lower than that of the control rabbits (at pre-cooling: 213 ± 10 vs. 275 ± 15 beats/min, *p* = 0.004) (Figure 3). Remimazolam at 0.1 mg/kg/h also lowered the heart rate; however, the difference was not statistically significant. Animals in the 0.1 and 1 mg/kg/h remimazolam showed lower mean arterial pressure than those in the control animals; however, the differences were not statistically significant (Tables 1, 2). The lactate levels were higher in the 1 mg/kg/h remimazolam group compared with the control group. Body weight, arterial pH, partial pressure of carbon dioxide and oxygen, base excess, HCO_3_, Na^+^, K^+^, Ca^2+^, glucose, hematocrit, and hemoglobin values did not differ significantly among the groups.

**TABLE 1:** Characteristics before cooling.

	Control	R 0.1	R 1	R 1 + F
N	6	6	6	6
Body weight (kg)	3.1 ± 0.0	2.9 ± 0.2	3.3 ± 0.1	3.2 ± 0.1
Core temperature (°C)	39.4 ± 0.2	38.7 ± 0.1	39.4 ± 0.2	38.8 ± 0.1
Heart rate (beats/min)	274 ± 15	247 ± 15	213 ± 10**	241 ± 17
Mean arterial pressure (mmHg)	81 ± 5	72 ± 5	74 ± 4	83 ± 4
Respiratory rate (breaths/min)	60 ± 4	66 ± 7	56 ± 6	63 ± 4
Arterial pH	7.48 ± 0.02	7.48 ± 0.03	7.43 ± 0.02	7.46 ± 0.02
PaCO_2_ (mmHg)	24.8 ± 1.9	26.2 ± 3.0	27.5 ± 3.3	33.1 ± 2.9
PaO_2_ (mmHg)	194 ± 9	171 ± 17	202 ± 12	188 ± 6
Base excess (mmol/L)	−5.3 ± 1.1	−3.8 ± 2.0	−5.5 ± 2.5	−0.5 ± 1.7
HCO_3_^−^ (mmol/L)	18.4 ± 1.1	19.4 ± 1.9	18.5 ± 2.2	23.6 ± 1.6
Na^+^ (mmol/L)	142 ± 2	144 ± 2	145 ± 1	144 ± 1
K^+^ (mmol/L)	3.7 ± 0.2	3.1 ± 0.1	3.1 ± 0.1	3.3 ± 0.2
Ca^2+^ (mmol/L)	1.7 ± 0.0	1.7 ± 0.0	1.7 ± 0.0	1.7 ± 0.1
Lactate (mmol/L)	4.9 ± 0.9	4.3 ± 0.8	11.1 ± 2.0*	5.3 ± 2.0
Glucose (mg/dl)	154 ± 12	187 ± 41	187 ± 24	147 ± 7
Hematocrit (%)	39.0 ± 1.4	38.3 ± 0.8	36.7 ± 1.0	37.8 ± 1.2
Hemoglobin (g/dl)	13.3 ± 0.5	13.0 ± 0.3	12.5 ± 0.3	12.9 ± 0.4

Data are expressed as mean ± SEM.

R 0.1, remimazolam 0.1 mg/kg/h; R 1, remimazolam 1 mg/kg/h; F, flumazenil.

PaCO_2_, partial pressure of carbon dioxide; PaO_2_, partial pressure of oxygen.

*: *p* < 0.05,**: *p* < 0.01 compared with the control group.

**TABLE 2:** Characteristics during shivering.

	Control	R 0.1	R 1	R 1 + F
N	6	6	6	6
Core temperature (°C)	38.4 ± 0.2	37.5 ± 0.4**	36.9 ± 0.1***	37.8 ± 0.1
Heart rate (beats/min)	247 ± 12	224 ± 16	207 ± 9	261 ± 16
Mean arterial pressure (mmHg)	83 ± 7	72 ± 6	80 ± 6	87 ± 5
Respiratory rate (breaths/min)	61 ± 5	61 ± 8	51 ± 4	55 ± 5
Arterial pH	7.51 ± 0.01	7.48 ± 0.03	7.43 ± 0.03	7.46 ± 0.02
PaCO_2_ (mmHg)	22.7 ± 1.4	26.0 ± 2.8	28.9 ± 1.5	30.1 ± 2.7
PaO_2_ (mmHg)	201 ± 10	185 ± 13	205 ± 15	199 ± 6
Base excess (mmol/L)	−4.8 ± 0.8	−5.7 ± 1.2	−7.0 ± 2.5	−2.3 ± 1.6
HCO_3_^−^ (mmol/L)	18.1 ± 0.9	18.2 ± 1.2	18.0 ± 2.0	22.7 ± 1.6
Na^+^ (mmol/L)	141 ± 1	147 ± 3	146 ± 2	144 ± 1
K^+^ (mmol/L)	3.3 ± 0.1	2.9 ± 0.2	3.2 ± 0.1	3.2 ± 0.2
Ca^2+^ (mmol/L)	1.7 ± 0.0	1.7 ± 0.1	1.7 ± 0.0	1.7 ± 0.1
Lactate (mmol/L)	5.7 ± 1.0	4.6 ± 0.9	11.9 ± 2.9*	5.5 ± 2.0
Glucose (mg/dl)	162 ± 15	181 ± 40	172 ± 18	143 ± 7
Hematocrit (%)	38.7 ± 1.6	36.8 ± 1.6	36.0 ± 1.3	37.7 ± 1.0
Hemoglobin (g/dl)	13.2 ± 0.5	12.5 ± 0.5	12.2 ± 0.4	12.9 ± 0.3

Data are expressed as mean ± SEM.

R 0.1, remimazolam 0.1 mg/kg/h; R 1, remimazolam 1 mg/kg/h; F, flumazenil.

PaCO_2_, partial pressure of carbon dioxide; PaO_2_, partial pressure of oxygen.

*: *p* < 0.05, **: *p* < 0.01, ***: *p* < 0.001 compared with the control group.

## Discussion

In the present study, we found that remimazolam administration at 1 mg/kg/h successfully suppressed shivering in rabbits. A selective GABA type A receptor antagonist, flumazenil, fully reversed the inhibitory effect of remimazolam. Moreover, 1 mg/kg/h remimazolam administration for 2 h significantly reduced the heart rate, which is clinically intuitive and consistent with the finding of a previous study in which remimazolam inhibited the cardiovascular function in a dose-dependent manner (Upton et al., 2009). Most of the hemodynamic and physiological parameters did not differ significantly among groups during cooling.

In the brain, GABA works as an inhibitory neurotransmitter and reduces neuronal excitability. The medial preoptic area in the hypothalamus is considered responsible for the thermoregulatory system in mammals (Nakamura and Morrison, 2010). Cold-sensory signals or systemic inflammation inactivates the inhibitory projection neurons in the medial preoptic area, including GABAergic neurons. As a result, the activation of somatic neurons and the consequent shivering occur through the efferent pathway (Nakamura and Morrison, 2010). Benzodiazepines bind to an extracellular site of the α and γ subunits of the GABA type A receptor and facilitate the inhibitory actions of GABA (Sigel and Ernst, 2018; Kim and Hibbs, 2021). Consequently, benzodiazepines modulate the central thermoregulatory system and lower the shivering threshold (Masamune et al., 2009). In the present study, we observed that the GABA type A antagonist flumazenil completely abrogated the anti-shivering effect of remimazolam, demonstrating that remimazolam exerted its anti-shivering effect through the GABA pathway.

Remimazolam is a novel benzodiazepine designed for rapid hydrolyzation and inactivation by nonspecific tissue esterases (Chitilian et al., 2013; Kilpatrick, 2021). Conventional benzodiazepines, such as midazolam, also have an inhibitory effect on shivering. However, these drugs are generally long-acting, and thus less useful in terms of controllability in the surgical settings due to the risk for prolonged respiratory suppression or sedative effects. Although flumazenil can be used as a reversal agent for conventional benzodiazepines, there is a concern for recurrent sedation due to the longer half-life [e.g., midazolam: 2–6 h (Greenblatt et al., 1984), diazepam: 24–57 h (Reves et al., 1985)] compared with that of flumazenil [approximately 1 h (Brogden and Goa, 1991)]. Furthermore, the inhibitory effect of conventional benzodiazepines on shivering is relatively weak, and they lower the shivering threshold by only small degrees. For example, even extremely high-dose midazolam (40 mg over the course of 4 h) reduced the shivering threshold by only 0.6°C (Kurz et al., 1995; Masamune et al., 2009). In contrast, we observed that low-dose remimazolam was sufficient to suppress shivering. Based on the animal–human dose conversion, the higher dose used in the present study (1 mg/kg/h) is equivalent to approximately 0.3 mg/kg/h in humans (Reagan-Shaw et al., 2008), which is clinically considered a low dose and is commonly used in everyday practice.

In the present study, remimazolam was discontinued 30 min prior to the initiation of cooling, and therefore the anesthetic effects of the ultrashort-acting remimazolam should already have disappeared. Indeed, patients usually recover from remimazolam anesthesia within 30 min after discontinuation without the need for flumazenil administration. Our findings indicate that remimazolam can suppress shivering even after its anesthetic effects disappear, which is of great clinical advantage. Remimazolam anesthesia may have the potential to prevent postoperative shivering without increasing the risk for remaining anesthetic effects and accompanying respiratory complications. As remimazolam is an ultrashort-acting benzodiazepine, its anesthetic effects disappear spontaneously and quickly. Therefore, based on the findings of our present study, adding flumazenil to reverse the anesthetic effects of remimazolam may not be recommended from the postoperative shivering-preventative perspective.

Another clinical implication from the present study is that remimazolam may also be useful during therapeutic hypothermia. Mild hypothermia (2–3°C reduction in tissue temperature) can provide considerable tolerance against ischemia and is proven beneficial during recovery from cardiac arrest (Bernard et al., 2002, 2002; Logue et al., 2007; Ohta et al., 2007; Lin et al., 2022). Remimazolam may have the potential to prevent shivering and consequent deleterious complications during therapeutic hypothermia.

This study has some limitations. We used relatively young male animals. Considering that a majority of patients undergoing surgery or therapeutic hypothermia are middle-aged or older, the anti-shivering effect of remimazolam observed in the present study should be tested in aged animals. Future studies should also include female animals with different menopausal statuses. Another limitation of this study is that it was conducted in rabbits, and thus the findings cannot be directly applicable to humans. In addition, although the respiratory rate was not significantly different among groups, rabbits that received 1 mg/kg/h of remimazolam plus flumazenil exhibited higher PaCO_2_ values, which potentially influenced the shivering threshold. Finally, although physiological mechanisms of the thermoregulatory system and benzodiazepine’s pharmacological mechanism of action have already been reported (Nakamura and Morrison, 2010; Sigel and Ernst, 2018), a lack of pathophysiological analyses at the molecular level remains a limitation of this study. More comprehensive studies using electrophysiological and pathophysiological analyses will confirm and provide further information about mechanisms underlying the anti-shivering effect of remimazolam observed in this study.

## Conclusion

Remimazolam at a clinically relevant dose suppressed shivering *via* the GABA pathway even 30 min after discontinuation. Remimazolam may prevent shivering in patients undergoing surgery or therapeutic hypothermia.

## Data Availability

The raw data supporting the conclusions of this article will be made available by the authors, without undue reservation.
